# Amyloidosis and Thoracic Aortic Disease: A Scoping Review

**DOI:** 10.3390/jcm15155817

**Published:** 2026-07-25

**Authors:** Vasiliki Androutsopoulou, Konstantinos Spanos, Ioanna Giannouka, Konstantinos Tzimkas-Dakis, Konstantinos Karakoussis, Athanasios D. Giannoukas

**Affiliations:** 1 Department of Cardiothoracic Surgery, University Hospital of Larissa, Faculty of Medicine, School of Health Sciences, University of Thessaly, 41222 Larissa, Greece; androutsopoulouvasiliki@hotmail.com; 2 Department of Vascular Surgery, University Hospital of Larissa, Faculty of Medicine, School of Health Sciences, University of Thessaly, 41222 Larissa, Greece; kostasdakis1994@gmail.com (K.T.-D.); agiannoukas@hotmail.com (A.D.G.); 3 Department of Internal Medicine, General Hospital of Larissa, 41222 Larissa, Greece; igiannouka@gmail.com (I.G.); karakoussis@yahoo.gr (K.K.)

**Keywords:** amyloidosis, thoracic aorta, aneurysm, dissection, AL amyloidosis, ATTR amyloidosis, aortopathy

## Abstract

**Background and Objectives**: Amyloidosis is a systemic disorder characterized by extracellular deposition of misfolded protein fibrils, most commonly light-chain (AL) or transthyretin-derived (ATTR) ones. Cardiac involvement is well recognized, but large-vessel complications, including thoracic aortic aneurysms (TAA) and dissections, are rare and under-reported. The aim of this review article is to provide insights into pathophysiology, clinical diagnosis and the therapeutic opportunities in amyloidosis-related thoracic aortic diseases. **Methods and Materials**: The PRISMA Extension for Scoping Reviews (PRISMA-ScR) Guidelines were followed. An extensive search of the available literature in the English language, published between 1 January 2000, and 31 December 2025, in three large-scale scientific databases was undertaken by two independent reviewers. The terms “amyloidosis”, “thoracic aorta”, “thoracic aortic aneurysm”, “aortic dissection”, and “aortopathy” were used both as specific items, as well as in MeSH Terms. Studies reporting on the pathophysiology, diagnosis, clinical manifestations, treatment options and prognosis of amyloid deposition on the thoracic aorta were included in the review. Because of the nature of the existing literature, only a narrative review was possible. **Results**: Twenty-nine studies were included. Owing to the rarity of reporting, data was derived mainly from case reports and series, as well as autopsy studies. Amyloid infiltration of the aortic wall has been associated with medial architectural disruption, degeneration of elastic fibers, impairment of vasa vasorum perfusion, and arterial stiffness, all of which could contribute to aneurysmal dilation and aortic lesions. Amyloidosis management combines targeted anti-plasma cell therapy with supportive care. In AL amyloidosis, melphalan–dexamethasone (MDex) was historically standard, but regimens such as cyclophosphamide, bortezomib, and dexamethasone (CyBorD) and bortezomib, melphalan, and dexamethasone (BMDex) achieve higher complete response rates. Immunotherapy with Daratumumab has shown high overall and complete response rates. Fibril-directed approaches, including doxycycline and epigallocatechin gallate, and monoclonal antibodies, are under evaluation. Acute management of large-vessel manifestations follows conventional protocols, but prognosis is often dominated by underlying cardiac and systemic involvement. **Conclusions**: Management of thoracic aortic involvement follows standard imaging surveillance and surgical criteria, though operative risk is increased. Multidisciplinary care, early recognition, and individualized risk stratification are essential to improve outcomes, particularly given frequent cardiac involvement.

## 1. Introduction

Amyloidosis is a systemic disorder characterized by extracellular deposition of misfolded protein fibrils, most commonly light-chain (AL) or transthyretin-derived (ATTR) ones. AL amyloidosis results from the deposition of misfolded immunoglobulin light chains produced by a clonal plasma cell disorder, characterized by rapid progression and multiorgan involvement. In contrast, ATTR amyloidosis results from the deposition of misfolded transthyretin, occurring either as an inherited disorder caused by pathogenic TTR variants, or as an age-related disease, with predominant cardiac and neurological manifestation [1,2]. Cardiovascular involvement may be a consequence of monoclonal light chains associated with plasma cell dyscrasias or, in the case of ATTR, may be age-related (wild type) or hereditary [1,2]. ATTR has been linked with aortic stenosis (AS) and heart failure in about 11% of cases having poor prognosis, although the disease remains largely underdiagnosed and the underlying pathophysiology remains dubious [1,2,3,4]. Wild-type transthyretin deposition has been increasingly recognized in elderly patients with severely calcified aortic valve stenosis, with reported prevalence estimates of approximately one in seven patients, in selected population cohorts [5]. Both ATTR cardiomyopathy and AS exhibit overlapping clinical features as well as several etiological parallels. ATTR has been linked with AS due to transthyretic deposition frequently identified within stenotic aortic valves in elderly patients. Although the precise associated mechanism remains incompletely understood, shared age-related degenerative mechanisms and local amyloid deposition could possibly contribute to the frequent coexistence of these conditions [5]. Emerging research indicates that oxidative stress, inflammation, and extracellular remodeling are key factors in the underlying pathology of both ATTR and AS [5].

Both subtypes of amyloidosis appear capable of compromising aortic integrity, though AL amyloidosis is more frequently associated with vascular complications due to aggressive systemic disease [6]. ATTR has not been typically recognized as a predisposing factor for thoracic aortic disease, including aneurysms and dissections, although sporadic case reports and autopsy studies suggest that amyloid infiltration of the aortic wall may compromise structural integrity, predisposing to aneurysmal dilation and dissection [6,7].

The aim of this review article is to provide insights on pathophysiology, clinical diagnosis and the therapeutic opportunities in amyloidosis-related thoracic aortic diseases as the understanding of this relationship is increasingly relevant given the aging population, rising prevalence of amyloidosis, and improved patient survival.

## 2. Materials and Methods

### 2.1. Eligibility Criteria

**Figure 1:**
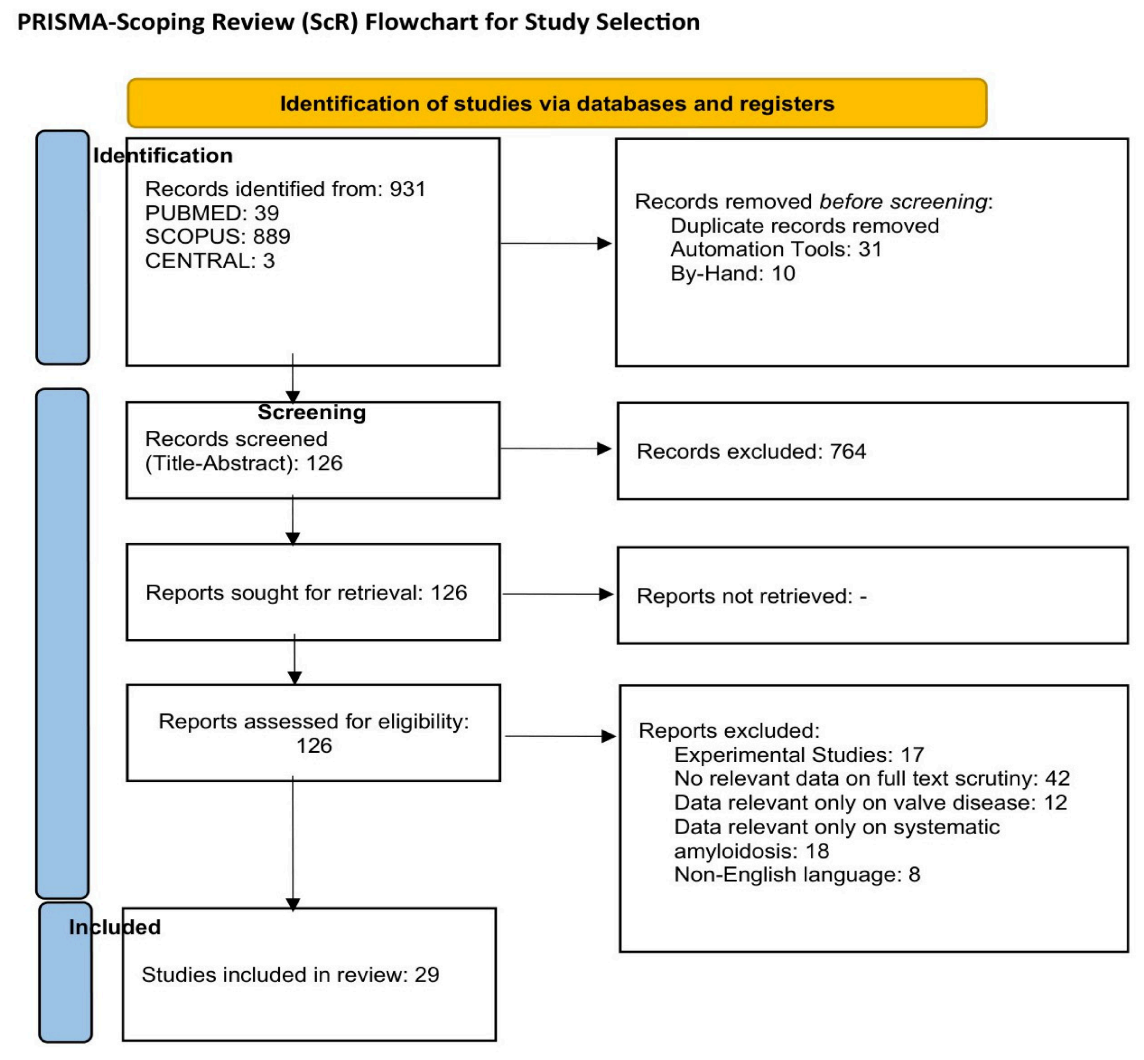
PRISMA ScR flowchart.

The PRISMA Extensions for Scoping Reviews (PRISMA-ScR) Guidelines were followed [8] (Figure 1). An extensive search of the literature in the English language on three large-scale databases (MEDLINE, SCOPUS, Cochrane CENTRAL) was conducted with a timeframe between 1 January 2000, until 31 December 2025. The PICO framework was applied for providing a clinically guided, structured search strategy [9] (Table 1). Studies reporting on amyloidosis-related thoracic aortic disease with a focus on pathophysiology, clinical management, treatment options and prognosis were included in the final analysis. Randomized and non-randomized studies, autopsy studies, case reports and series, and translational analyses were to be included in the final review. Non-English-language and experimental animal studies were excluded.

### 2.2. Search Strategy

The terms “amyloidosis”, “thoracic aorta”, “aneurysm”, “dissection”, and “aortopathy” were used in various combinations, both as specific items, as well as MeSH Terms. (Table 2). Due to the recognized role of medin as the most prevalent, age-associated vascular amyloid, as well as its emerging association with the degeneration of the thoracic aorta, studies specifically examining medin amyloidosis in the context of thoracic aortic lesions were included in the review. Duplication screening was carried out via both automated tools [EndNote 20.2.1 X9, Clarivate (Philadelphia, PA, USA)] and via manual inspection of all studies produced at the initial search. An initial selection of relevant studies was undertaken by two independent reviewers (V.A, K.S.), and a secondary selection process was performed according to the full text. Discrepancies at any step of the process were resolved after discussion with a third investigator (A.G.) A standard title, abstract, and full-text evaluation of the included studies was undertaken, including tables and figures of relevant studies. References for the included studies were furthermore checked for possibly missed relevant studies. 

Total Hits: 931

Duplicate Screening:

Automatic Tool Duplicate Removal: 900 (31 duplicates excluded)
By-Hand Duplicate Removal: 890 (10 duplicates excluded)

Title-Abstract Screening: 126 (764 excluded)

Full-text Screening: 29 (97 excluded)

### 2.3. Data Extraction and Analysis

Data extraction was executed on a standard Microsoft Excel spreadsheet. Applicable data on pathophysiology, clinical relevance, conservative and invasive treatment strategies and findings on patient prognosis were included. Due to the nature of the existing literature, only a narrative review was possible and finally completed.

## 3. Ethics

The current review complied with the declaration of Helsinki principles. Scientific Council approval was not required.

## 4. Results

### 4.1. Study Selection

The initial search yielded 931 studies across all three databases. Following duplicate removal, 41 studies were excluded (31 via automated duplicate removal tools, 10 via manual duplicate removal). Following title and abstract screening, 764 studies were excluded. Full-text analysis excluded 97 studies, ultimately producing 29 studies for review. Studies’ characteristics, design, objective and principal findings relevant to thoracic aortic disease can be found in Table 3.

#### 4.1.1. Amyloid Deposition in the Thoracic Aorta and Related Pathophysiology

Arterial amyloidosis refers to the deposition of amyloidogenic proteins within the arterial wall. To date, up to 30 distinct amyloid proteins or peptides have been identified in humans. These proteins are characterized by their β-sheet secondary structure, fibrillar morphology with diameters ranging from 7 to 13 nm, Congo red positivity (appearing red), and apple-green birefringence when viewed under polarized light microscopy [10].

Oxidative stress, chronic inflammation, as well as extracellular matrix remodeling are known to represent common biological pathways in both amyloidosis and thoracic aortic disease [3,5,10]. Oxidative stress promotes protein misfolding and amyloid fibril formation, while contributing to endothelial dysfunction and vascular smooth muscle cell injury [3,5]. Persistent inflammatory signaling further amplifies tissue damage through cytokine release, macrophage activation and increased expression of matrix-metalloproteinases, particularly MMP-2. As a result, a degradation of elastin and collagen within the aortic media is observed [7,10]. These extracellular matrix alterations promote medial degeneration, increased arterial stiffness, and progressive aortic wall degeneration, all changes which have been detected in thoracic aortic aneurysm formation and dissection [7,10,14]. The abovementioned mechanisms have been investigated in medin-associated vascular amyloidosis, potentially providing a plausible framework for understanding how amyloid deposition could contribute to thoracic aortic lesions in systemic AL and ATTR amyloidosis [3,5,10,11]. However, direct clinical evidence is limited.

Medin is a 50-amino acid peptide derived from milk fat globule EGF-like factor 8 protein (MFGE8), and it is the most prevalent human amyloid, occurring in about 97% of individuals over 50 years old. Medin is recognized as the most prevalent amyloidogenic protein in the upper arterial system observed in aged aortic and cerebrovascular walls in humans. It demonstrates a strong affinity for elastic fibers and is closely linked with arterial degenerative inflammation, elastic fiber fragmentation, calcification, and amyloidosis [10]. Both medin and ATTR are age-associated amyloid proteins which have been detected in the aortic wall. Mechanistic studies of medin provide crucial insights into amyloid-associated vascular degeneration. However, the extent to which these mechanisms apply directly to AL/ATTR-related thoracic aortic lesions remains uncertain. Analyses using immunostaining and immunoblotting of human samples reveal that medin levels are elevated during the pathogenesis of aortic aneurysm and dissection [10].

It is widely recognized that idiopathic degenerative thoracic aneurysms display increased concentrations of amyloid oligomers as well as changes in the micromechanical and structural features of the vessel wall [11]. These findings seem to support a plausible, from a biological standpoint, role for amyloid-mediated aortic degeneration. Nevertheless, direct evidence providing a link between these mechanisms and systemic AL or ATTR amyloidosis remains limited. Previous studies have documented increased expression of matrix metalloproteinase 2 (MMP2) following the introduction of medin to cells. Elevated MMP2 activity has also been associated with thoracic aortic aneurysm and dissection. Most patients with degenerative aneurysms exhibit oligomeric aortic medial amyloid, which may play a significant role in thoracic aortic aneurysm (TAA) pathogenesis [11]. In contrast, this phenomenon is not observed in patients with bicuspid aortic valve (BAV), suggesting that amyloid deposition is not a contributing factor in BAV-associated aortopathy [11]. Despite these mechanistic observations, clinical evidence linking amyloid deposition to thoracic aortic disease remains limited. Consequently, the true prevalence and clinical significance of thoracic aortic involvement in systematic amyloidosis remains uncertain, while increased clinical awareness may facilitate earlier recognition in some patients [11].

**Figure 2:**
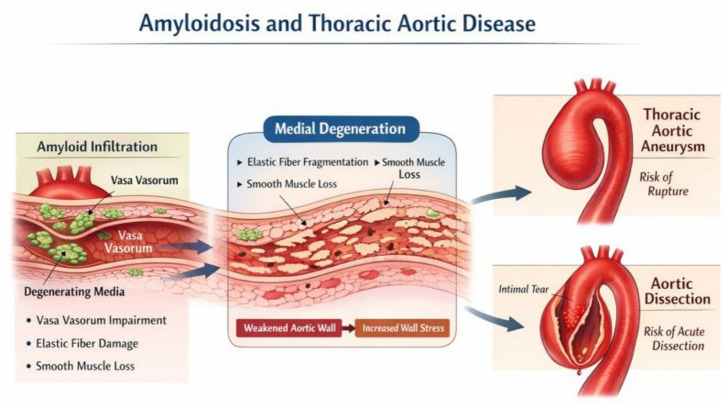
Schematic illustration of amyloid infiltration of the thoracic aortic wall. Established histopathological findings include medial degeneration (elastic fiber fragmentation, smooth muscle fiber loss), vasa vasorum involvement, and arterial stiffening. The proposed progression to aneurysm formation and aortic dissection represents a hypothetical mechanism based on limited clinical and experimental evidence.

Amyloid infiltration of the thoracic aortic wall causes disruption of the elastic fibers and reduction in smooth muscle cell density in the media and involvement of the vasa vasorum in the adventitia, impairing wall nutrition [11]. Finally, it may weaken the mechanical integrity of the elastic lamellae, resulting in fragmentation (Figure 2).

All these changes result in medial degeneration, arterial stiffening with loss of elasticity, which increases pulse wave velocity and wall stress, and reduced microvascular perfusion, promoting ischemic medial degeneration, and all the aforementioned mechanisms in synergy with the conventional risk factors such as hypertension, advanced age, and atherosclerosis exacerbate the structural vulnerability predisposing the aorta to dilate with aneurysm formation or dissection [3,4,12,13,14].

#### 4.1.2. Clinical Evidence and Diagnosis

The clinical evidence is weak and is largely derived from case reports, small series, and autopsy studies [6,7,15,16]. Thoracic aneurysms are sparsely reported in both AL and ATTR amyloidosis, often as incidental findings on imaging or postmortem examination. In a National Inpatient Sample analysis of hospitalized patients with thoracic aortic disease, concomitant amyloidosis was found in approximately 0.2% of patients. AL amyloidosis was detected more commonly than ATTR, while affected patients were of younger age, compared to those with ATTR amyloidosis [16]. Thoracic dissections have also been reported as sporadic cases in patients with systemic amyloidosis, sometimes presented atypically due to concurrent cardiac dysfunction [6,7,16,17]. Clinical suspicion should always be raised in patients with known systemic amyloidosis, particularly those with cardiac involvement, advanced age, or additional risk factors [16,17]. CT angiography and MRI remain standard for identifying TAA, aortic wall abnormalities, and dissections. As systemic amyloidosis is more common than localized amyloidosis associated with serious implications, it is of note that in systemic amyloidosis and organ dysfunction there are some common proteins involved such as immunoglobulin (Ig) LCs (AL), Ig heavy chain (AH), transthyretin (ATTR), serum amyloid A (AA), apolipoprotein A-I (AApoAI), β2-microglobulin, and leukocyte chemotactic factor-2 (ALECT2) [18]. Additionally, over the last decade the adoption of biomarker-based screening for AL amyloidosis in patients with monoclonal gammopathy of undetermined significance has been encouraged, as some available biomarkers such as N-terminal pro-brain natriuretic peptide (NT-proBNP) have improved diagnostic accuracy and risk stratification [19] in cardiac amyloid and albuminuria in renal amyloid [20]. It is important to note that amyloid deposition in the aortic wall is frequently recognized in histopathology only after surgical repair or autopsy, suggesting underdiagnosis. Congo red staining with birefringence under polarized light confirms amyloid deposition [4,17,21].

#### 4.1.3. Management and Prognosis

The medical management of amyloidosis involves both targeted therapies aimed at mitigating amyloid deposition and general measures designed to prevent disease-related complications. Melphalan-dexamethasone (MDex) was the standard-of-care upfront treatment for AL amyloidosis [20]. Also, cyclophosphamide, bortezomib, and dexamethasone (CyBorD) combination, and bortezomib, melphalan, and dexamethasone (BMDex) combination, showed better complete response as compared to only MDex treatment [23,24]. Immunotherapy with Daratumumab has recently been evaluated in patients with AL amyloidosis, associated with a remarkable overall response rate (ORR) of 76% and a complete response rate (CRR) of 36% [25]. Additionally, fibril-directed therapies with doxycycline and epigallocatechin gallate, as well as the use of monoclonal antibodies, have been associated with some promising results [26,27,28,29,30] (Table 4).

The standard principles of thoracic aortic disease should be followed with serial imaging to monitor aneurysm progression. A multidisciplinary approach remains essential, requiring coordination among cardiology, vascular/cardiothoracic surgery, anesthesiology, and hematology. Surgical intervention is indicated for symptomatic aneurysms or when size criteria are met. The operative risk is higher due to tissue fragility, bleeding tendency, and concomitant cardiomyopathy. Mortality is often driven by cardiac involvement rather than aneurysm or dissection. Acute management of dissection follows conventional protocols, but prognosis is often dominated by underlying cardiac and systemic involvement [21,31].

## 5. Discussion

The overall outcomes in amyloidosis-related thoracic aortic disease are poorer than in conventional aortopathy [16,32]. Management of thoracic aortic disease in patients with systemic amyloidosis requires a careful balance between addressing the underlying plasma cell disorder and mitigating the unique cardiovascular risks posed by amyloid infiltration. Early recognition, risk stratification, and individualized management improve perioperative safety and long-term outcomes. Historically, melphalan–dexamethasone (MDex) was the mainstay of treatment for AL amyloidosis, but combination regimens such as cyclophosphamide, bortezomib, and dexamethasone (CyBorD) and bortezomib, melphalan, and dexamethasone (BMDex) have demonstrated superior complete response rates, potentially reducing systemic amyloid burden and improving overall cardiovascular stability [22,23,24]. More recently, Daratumumab, an anti-CD38 monoclonal antibody, has shown remarkable efficacy, with an overall response rate of 76% and complete response in 36% of patients [26], highlighting the potential of immunotherapy to alter disease trajectory.

Beyond systemic therapy, fibril-directed interventions such as Doxycycline and epigallocatechin gallate, as well as emerging monoclonal antibodies, offer promising adjunctive strategies to directly target amyloid deposits [26,27,28,29,30]. For thoracic aortic involvement, standard surveillance with serial imaging remains critical, as amyloid infiltration can exacerbate aortic wall fragility, increase operative risk, and accelerate aneurysm progression. Surgical intervention is indicated based on conventional size or symptomatic criteria, yet outcomes are often dominated by cardiac amyloid involvement, underscoring the need for a multidisciplinary approach integrating cardiology, hematology, and cardiothoracic surgery. Ultimately, current treatment options for AL amyloidosis include plasma cell-directed therapies as well as fibril-targeting approaches, which have substantially improved hematologic outcomes. However, evidence demonstrating a direct effect of these therapies on thoracic aortic involvement is currently lacking.

Prospective studies are needed to determine the prevalence and natural history of amyloid-related thoracic aortic disease. The development of noninvasive imaging biomarkers to detect amyloid deposition in the aortic wall at an early stage will be of paramount importance. Artificial intelligence may assist in the development of risk stratification models for thoracic aortic aneurysm and dissection [33]. While contemporary diagnostic tools for thoracic aortic pathologies are mainly CT and MR angiography, it should be guided by clinical suspicion in patients with known systematic amyloidosis, in addition to heart involvement and other known risk factors. Finally, potential investigation of disease-modifying therapies for systemic amyloidosis targeting transthyretin or amyloid protein may be able to reduce vascular complications. The principal goal is to develop anti-fibril therapies capable of safely and effectively removing amyloid deposits, ultimately restoring the normal function of affected organs.

All available evidence suggesting a link between amyloidosis and thoracic aortic lesions, including pathophysiological involvement, diagnostic pathways, therapeutic interventions and follow-up strategies, should be interpreted cautiously. The true prevalence, natural history, and clinical impact of thoracic involvement in systematic amyloidosis remain uncertain, without any robust data for a causal relationship to be established. Future studies should clarify the prevalence, natural history, and clinical importance of thoracic aortic lesions in amyloidosis, and determine whether disease-modifying therapies could influence the progression of amyloid-associated aortopathy.

## 6. Limitations

The current review bears some important limitations. The available evidence is derived predominantly from case reports, small case series, autopsy studies and observational analyses, thus limiting the ability to establish causality between amyloidosis and thoracic aortic lesions. Considerable heterogeneity exists regarding the amyloid subtypes, study population characteristics, diagnostic methods for detection of lesions, and reported outcomes. Publication and reporting bias are also considerable, particularly due to the rarity of clinically significant cases which are ultimately reported. The scarcity and heterogeneity of the available data preclude quantitative synthesis and analyses. The abovementioned limitations allow for a descriptive analyses of data, which should be cautiously interpreted.

## 7. Conclusions

Amyloidosis may rarely involve the thoracic aorta and may contribute to aneurysmal degeneration and dissection, although current available evidence remains limited. Amyloid infiltration compromises medial integrity, increases arterial stiffness, and weakens the aortic wall, particularly in the presence of conventional risk factors. Awareness of this association is essential for diagnosis, surveillance, and surgical planning, with multidisciplinary management optimizing patient outcomes. Clinicians should always maintain a high index of suspicion on the grounds of thoracic pain, unexplained aortic dilatation, or other imaging abnormalities of the thoracic aorta. In such patients, contrast-enhanced CT or MR angiography should be performed according to current thoracic aortic disease guidelines and recommendations. Multidisciplinary evaluation, including cardiology, hematology, and aortic specialists, is advised to guide individualized treatment.

## Figures and Tables

**Table 1:** PICO model.

P	Patient, population or problem	Patients with thoracic aortic disease due to amyloid deposition in the aorta
I	Intervention, prognostic factor or exposure	Any kind of conservative or invasive treatment
C	Comparison of intervention	-
O	Outcome you would like to measure or achieve	-
	What type of question are you asking?	-What are the pathophysiology processes encountered in amyloid deposition on the thoracic aorta? -What is the clinical significance and presentation of amyloid deposition on the thoracic aorta? -What does the diagnostic pathway for patients with thoracic aortic disease due to amyloid deposition include? -What is the management and prognosis of patients with amyloid-related thoracic aortic disease?
	Type of study you want to find	Observational studies

**Table 2:** Search strategy per protocol.

PUBMED Hits: 39	((amyloidosis) OR (amyloidosis[MeSH Terms])) AND (((((thoracic aorta) OR (thoracic aorta[MeSH Terms])) OR ((aortopathy) OR (aortopathy[MeSH Terms]))) OR ((thoracic aortic aneurysm) OR (thoracic aortic aneurysm[MeSH Terms]))) OR ((aortic dissection) OR (aortic dissection[MeSH Terms])))
SCOPUS Hits: 889	“amyloidosis” AND “thoracic aorta” OR “thoracic aortic aneurysm” OR “aortic dissection” OR “aortopathy”
CENTRAL Hits: 3	#1 amyloidosis #2 [mh “amyloidosis”] #3 thoracic aorta #4 [mh “thoracic aorta”] #5 thoracic aortic aneurysm #6 [mh “thoracic aortic aneurysm”] #7 aortic dissection #8 [mh “aortic dissection”] #9 aortopathy #10 [mh “aortopathy”] #11 {OR #1–#2} #12 {OR #3–#10} #13 {AND #11–#12}

**Table 3:** Included studies’ design, objective and principal finding relevant to thoracic aortic disease.

Ref.	First Author	Year	Study Design	Study Objective	Principal Finding Relevant to Thoracic Aortic Disease
[3]	Falk et al.	2016	Review	To review the pathophysiology, diagnosis, and treatment of AL cardiac amyloidosis.	Overview of AL amyloidosis pathophysiology and cardiovascular involvement.
[4]	Maleszewski et al.	2015	Review	To summarize the pathology, nomenclature, and typing of cardiac amyloidosis.	Histopathological features and typing of amyloid deposition.
[5]	Maerivoet et al.	2025	Review	To review the interaction between medin and transthyretin in the aortic wall and valves.	Medin and ATTR may synergistically contribute to vascular degeneration.
[6]	Choudhury et al.	2025	Case report	To describe a rare case of isolated AL amyloid involvement of the aortic valve.	Demonstrates cardiovascular amyloid deposition involving the aortic valve.
[7]	Peng et al.	2007	Experimental study	To investigate the role of medin in thoracic aortic aneurysm pathogenesis.	Medin promotes MMP-2 activation and medial degeneration associated with thoracic aneurysms.
[10]	Wang et al.	2023	Review	To summarize the role of medin in arterial aging and vascular disease.	Medin contributes to age-related arterial remodeling and vascular amyloidosis.
[11]	Davies et al.	2019	Histopathological study	To investigate medial amyloid deposition in degenerative thoracic aneurysms.	Degenerative thoracic aneurysms are associated with increased medial amyloid deposition.
[12]	Okada et al.	2023	Observational study	To evaluate transthyretin amyloid deposition in cardiovascular tissues.	ATTR deposition supports potential vascular involvement.
[13]	Hoteit et al.	2024	Case report	To report wild-type ATTR deposition in an ascending aortic aneurysm.	ATTR was identified within aneurysmal aortic tissue.
[14]	Nemes et al.	2018	Observational study	To assess aortic stiffness in patients with cardiac amyloidosis.	Cardiac amyloidosis is associated with increased aortic stiffness.
[15]	Ibrahimi et al.	2025	Case report	To describe the association between amyloidosis and degenerative arterial aneurysms.	Suggests a possible relationship between amyloidosis and aneurysmal disease.
[16]	Ersoezlue et al.	2025	National database study	To investigate the prevalence and characteristics of amyloidosis in patients with aortic disease.	Concomitant amyloidosis and aortic disease was uncommon (~0.2%).
[17]	Vrana et al.	2009	Diagnostic study	To evaluate proteomic typing of amyloid deposits using mass spectrometry.	Mass spectrometry accurately classifies amyloid subtype.
[18]	Sipe et al.	2016	Consensus statement	To update the nomenclature and classification of amyloid fibril proteins.	Standardized amyloid classification for clinical and research use.
[19]	Palladini et al.	2003	Cohort study	To evaluate NT-proBNP as a biomarker in AL amyloidosis.	NT-proBNP is a sensitive marker of cardiac involvement.
[20]	Palladini et al.	2014	Cohort study	To develop a renal staging system for AL amyloidosis.	Introduced renal staging and response markers.
[21]	Garcia-Pavia et al.	2021	Position statement	To provide recommendations for the diagnosis and treatment of cardiac amyloidosis.	Summarizes contemporary diagnostic and therapeutic strategies.
[22]	Palladini et al.	2007	Cohort study	To evaluate long-term outcomes following melphalan–dexamethasone therapy.	MDex produced durable hematologic responses.
[23]	Palladini et al.	2014	Matched case–control study	To compare MDex with bortezomib-containing regimens.	Bortezomib improved hematologic response rates.
[24]	Cibeira et al.	2015	Review	To review the role of CyBorD as first-line therapy for AL amyloidosis.	Supports CyBorD as an effective upfront treatment.
[25]	Kaufman et al.	2017	Clinical study	To evaluate Daratumumab in refractory AL amyloidosis.	Rapid and deep hematologic responses were observed.
[26]	Ward et al.	2011	Experimental study	To investigate doxycycline as an anti-fibril therapy.	Doxycycline reduced amyloid fibril formation.
[27]	Wechalekar et al.	2017	Clinical study	To evaluate the clinical impact of doxycycline in AL amyloidosis.	Doxycycline was associated with reduced early mortality.
[28]	Mereles et al.	2010	Clinical study	To investigate epigallocatechin gallate in cardiac AL amyloidosis.	Suggested potential benefit as adjunctive therapy.
[29]	Gertz et al.	2016	Phase I/II clinical trial	To evaluate NEOD001 in patients with persistent organ dysfunction due to AL amyloidosis.	Demonstrated preliminary safety and potential therapeutic benefit.
[30]	Edwards et al.	2017	Phase I clinical study	To evaluate a fibril-reactive monoclonal antibody (11-1F4).	Early clinical evaluation demonstrated promising safety and activity.
[31]	Milewicz et al.	2019	Review	To summarize current therapies for thoracic aortic aneurysm and dissection.	Provides contemporary management principles for thoracic aortic disease.
[32]	Gerra et al.	2025	Observational study	To assess the impact of amyloidosis on cardiovascular outcomes after valve intervention.	Amyloidosis adversely affected cardiovascular outcomes.
[33]	Kong et al.	2025	Review	To review artificial intelligence applications in cardiac amyloidosis.	AI may improve prognostic assessment and risk stratification.

**Table 4:** Therapeutic inhibitors and targeted agents’ mechanism of action, principal effect and evidence in thoracic aortic disease.

Therapeutic Class	Representative Agents	Mechanism of Action	Principal Effect	Evidence in Thoracic Aortic Disease
Plasma cell-directed therapy	Melphalan, Cyclophosphamide, Bortezomib, Dexamethasone	Eliminates the clonal plasma cells responsible for producing amyloidogenic light chains	Reduces or suppresses amyloid precursor protein production (AL amyloidosis)	No direct evidence
Anti-CD38 monoclonal antibody	Daratumumab	Targets CD38-expressing plasma cells, leading to plasma cell depletion	Reduces light-chain production (AL amyloidosis)	No direct evidence
Amyloid fibril inhibitors	Doxycycline, Epigallocatechin gallate (EGCG)	Inhibit amyloid fibril formation and promote fibril destabilization	Reduces amyloid deposition (investigational)	No direct evidence
Amyloid removal therapy	NEOD001, 11-1F4	Monoclonal antibodies targeting deposited amyloid fibrils to facilitate immune-mediated clearance	Promotes removal of existing amyloid deposits (investigational)	No direct evidence

## Data Availability

The original contributions presented in this study are included in the article. Further inquiries can be directed to the corresponding author.
